# Determinants of Change in Physical Activity in Children and Adolescents: A Systematic Review

**DOI:** 10.1016/j.amepre.2011.02.025

**Published:** 2011-06

**Authors:** Christopher Craggs, Kirsten Corder, Esther M.F. van Sluijs, Simon J. Griffin

**Affiliations:** MRC Epidemiology Unit, Institute of Metabolic Science and UKCRC Centre for Diet and Activity Research (CEDAR), Addenbrooke's Hospital, Cambridge, United Kingdom

## Abstract

**Context:**

Data are available on correlates of physical activity in children and adolescents, less is known about the determinants of change. This review aims to systematically review the published evidence regarding determinants of change in physical activity in children and adolescents.

**Evidence acquisition:**

Prospective quantitative studies investigating change in physical activity in children and adolescents aged 4–18 years were identified from seven databases (to November 2010): PubMed, SCOPUS, PsycINFO, Ovid MEDLINE, SPORTDdiscus, Embase, and Web of Knowledge. Study inclusion, quality assessment, and data extraction were independently validated by two researchers. Semi-quantitative results were stratified by age (4–9 years, 10–13 years, and 14–18 years).

**Evidence synthesis:**

Of the 46 studies that were included, 31 used self-reported physical activity; average methodologic quality was 3.2 (SD=1.2), scored 0–5. Of 62 potential determinants identified, 30 were studied more than three times and 14 reported consistent findings (66% of the reported associations were in the same direction). For children aged 4–9 years, girls reported larger declines than boys. Among those aged 10–13 years, higher levels of previous physical activity and self-efficacy resulted in smaller declines. Among adolescents (aged 14–18 years), higher perceived behavioral control, support for physical activity, and self-efficacy were associated with smaller declines in physical activity.

**Conclusions:**

Few of the variables studied were consistently associated with changes in physical activity, although some were similar to those identified in cross-sectional studies. The heterogeneity in study samples, exposure and outcome variables, and the reliance on self-reported physical activity limit conclusions and highlight the need for further research to inform development and targeting of interventions.

## Context

Higher levels of physical activity in childhood are associated with favorable metabolic and cardiovascular disease risk profiles,^1^ increased well-being,^2^ and normal skeletal development.^3^ However, levels of physical activity in children and adolescents remain a public health concern^4,5^ and have been shown to decline as children progress through childhood to adolescence.^6^ Thus, public health efforts are increasingly directed toward promoting physical activity and preventing this age-related decline.^7^ Physical activity promotion interventions have however met with limited success to date.^8^

To date, a broad range of factors has been investigated, including demographic, biological, environmental, social, and psychological. Several in-depth reviews focusing on correlates of physical activity in youth have also been published.^9–11^ Gender, age, SES, and parental and peer influences were among the most-researched correlates. However, previously identified correlates mostly relate to cross-sectional differences in levels of physical activity. Findings are therefore limited to hypothesis generation concerning potential causal factors and mediators.^12–14^

Understanding of factors associated with physical activity would be significantly enhanced by examination of these previously identified correlates, and other factors, in longitudinal studies. Identifying determinants—potential causal factors^12^—and mediators of change in child and adolescent physical activity should strengthen the evidence base to inform the development and targeting of effective interventions.^15^ Further, analyzing potential causal factors allows researchers to test the fit and utility of existing behavioral theories.^16^ As public health efforts focus on changing physical activity behaviors among children and adolescents,^7^ research may increasingly focus on change in physical activity behavior and its determinants. However, comparatively few studies have investigated determinants of change in physical activity, and no review has so far attempted to synthesize this evidence. Following the ecologic model of physical activity behavior,^17^ a systematic review was conducted of studies investigating potential determinants of change in physical activity in children and adolescents. The aim of the review is to collate the current evidence base, highlight research trends and limitations in physical activity determinants research, and synthesize the existing evidence.

## Evidence Acquisition

### Search Methods/Identification of Studies

Computer searches for reports of studies investigating determinants of change in physical activity in children and adolescents were conducted using seven electronic databases (PubMed, PsycINFO, SCOPUS, Embase, Ovid MEDLINE, Web of Knowledge, and SPORTDiscus) including all electronically archived literature within the databases up until November 2010. The search strategy was based on the study population, physical activity behavior and its longitudinal patterns, study design, and the investigation of determinants of change in physical activity.

### Inclusion/Exclusion Criteria

This review was restricted to studies published in English. To be included, a study had to be a prospective study quantifying change in physical activity in children or adolescents, and assessing at least one potential determinant of change. All studies were required to include a measure of physical activity at baseline and at follow-up. In addition, participants had to be within the range of 4–18 years within their measurement periods. Intervention studies were included only if a cohort analysis assessing associations between potential determinants and change in physical activity was reported.

All types of overall physical activity domains were included, except for studies focusing on a single specific behavior, such as active transport. As heterogeneity in change in the different domains of physical activity was anticipated, an a priori decision not to stratify by domain of physical activity was made. This review considers both determinants and longitudinal correlated changes in potential determinants and physical activity. Bauman et al.^12^ define a determinant as a preceding, causal predictor of change in physical activity. Results for determinants and associations between changes in physical activity and changes in the determinants were grouped together.

All studies identified through the database searches were extracted into an Endnote database. The titles, abstracts, and full texts of these papers were then screened for the inclusion criteria. The initial search and scanning was conducted by one reviewer and a 15% random sample was double checked at each title, abstract, and full-paper review stage, respectively. Should there have been a difference in opinions of more than one fifth of the doubly checked sample, further checks would have been completed. In four cases of differences in opinion, a consensus was reached by discussion or after consultation with a mediator. Reference lists of all papers included in the final sample were scanned for any additional relevant papers.

### Data Extraction

Data extraction for all included studies was undertaken using standardized forms by one reviewer, and independently validated by a review of two random 15% samples of the included papers. Any discrepancies were resolved by discussion. The extracted data included first author, publication year, title, journal, country, study population, study setting, baseline descriptive data, physical activity measurement, analysis method, length of follow-up, number of follow-up measurements and results. Where possible, results from adjusted multivariate models were extracted instead of single variable model results. In line with previously published systematic reviews, potential determinants were categorized as biological and demographic, sociocultural, psychological, or physical environment variables following previous research.^10^ Semiquantitative results were stratified into three groups according to the mean age of the study samples: 4–9 years, an age group covering the transitional period between ages 10–13 years and 14–18 years.

The a priori decision to stratify according to age was based on two main factors. First, correlates of physical activity have previously been shown to differ for children and adolescents^10,11^; thus, determinants of change may also differ according to age. Second, research has also suggested an impact of major life transitions on behavior change throughout the life course.^18^ One of these transitions may be from primary to secondary school, occurring approximately between ages 10 and 13 years. Publications that did not report a mean age for the sample population were categorized into age groups according to the middle value of the reported age range.

### Assessment of Methodologic Quality

A scale assessing methodologic quality was constructed (shown in Appendix A, available online at www.ajpm-online.net) and modified from previously reported checklists.^19^ The scale was focused on internal and external validity and all studies were assessed against the scale by one reviewer and independently validated by two random 15% samples of the included studies. The five-item scale is shown in Appendix A (available online at www.ajpmonline.org). Items were marked “positive,” “negative,” or “not sufficiently described.” A total score was calculated by adding all positive scores for each assessed study. The scoring system placed an emphasis on positive scores. Negative and not sufficiently described items were treated equally in that no points were scored for either.

### Strength of Evidence

Results supported by objective measures of physical activity and studies with higher methodologic quality were highlighted. The smallest individual subsample was considered as the unit of analysis.^20^ For instance, if results were stratified by boys and girls, two samples marked “m” for boys and “f” for girls were reviewed.

Because of the expected heterogeneity in a number of key aspects of the included studies—such as the constructs used to measure the exposure variables, type of physical activity measure used, length of follow-up, setting, and study population—an a priori decision not to meta-analyze the data was made. Instead, a classification system similar to previous systematic reviews^9–11^ was used. Significant associations (*p*<0.05) were noted as (++) or (– –), according to the direction of the association, whereas statistical findings below a threshold *p*-value <0.1 were reported as (+) and (–) for a positive or negative direction of association, respectively. Significant associations (*p*<0.05) without a stated direction of association were followed up by correspondence with the author; in case of no reply, the most likely direction of association was reported with reference to existing research. No association and inconclusive evidence were denoted by a (0) and (?), respectively. For a conclusion to be drawn, a determinant had to be reported by at least three study samples, and at least two thirds of the reported associations were required to be in the same direction.^10^ A positive, negative, or null association was reported as ++, – –, or 00 respectively. If it was not possible to reach a conclusion, an indeterminate association was reported as ??.

## Evidence Synthesis

**Figure 1:**
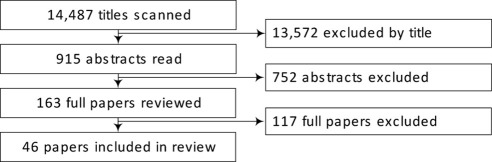
Flow of studies through the review process

Of 14,487 studies identified through all database searches, 163 papers were read in full and 46 papers were included (Figure 1). Potential papers were most commonly excluded because they did not address determinants of change in physical activity, examined cross-sectional data, or the sample population age did not match the review inclusion criteria (Table 1 and Appendix B [available online at www.ajpmonline.org] show descriptive summaries of all included studies^6,18,21–64^). Thirty-eight^6,21,23–36,38,39,41–44,47–58,60,62–64^ of all included studies were published after the year 2000, 30 studies^6,18,21,22,25,26,28,29,34,35,37–39,41–44,46–48,50–55,58,59,61,63^ were conducted in North America, and 13 studies^23,24,25,29,30,32,36,37,41,43,47,49,54^ reported a baseline sample size of more than 1000 participants.

For the majority of studies,^18,22–27,31–33,35,37,42,43,47–52,54,56–59,63^ the mean age of the sample population fell into a transitional age group between 10 and 13 years. Slightly fewer studies^28,30,34,36,38–41,45,53,55,60,64^ investigated adolescents aged 14-18 years, which largely included all-girl samples. A smaller number of studies^6,21,24,29,31,44,46,61,62^ reported findings for children aged 4–9 years. Of all included studies, 31^18,21,22,25–27,29,30,34,36–41,43–45,47–50,52–59,64^ utilized self-report measurements of physical activity, with six studies^6,23,32,46,51,61^ employing objective methods such as accelerometry or heart rate monitoring. A further nine studies^24,28,31,33,35,42,60,62,63^ combined objective with self-report measures of physical activity. All studies employing objective measures of physical activity were conducted in either the child or transitional age groups. No studies in the adolescent age group utilized objective measures. Mean methodologic quality for all studies was 3.2 (SD=1.2). Table 2 reports a summary of all associations between potential determinants and change in physical activity for all three age groups.^6,18,21–64^

### Children Aged 9 Years and Younger

Two of nine studies reported sample ages close to the cut-off point of >10 years.^29,61^ Nine studies^6,21,23,28,30,41,55,60,61^ investigated 26 biological and demographic, social, and psychological factors and their association with change in physical activity. Gender was consistently associated with change in physical activity: Girls exhibited larger declines than boys.^6,21,24,61^ Parental marital status was consistently shown not to be associated with change in physical activity.^44,61^ All other factors were classified as indeterminate associations.

### Children Aged 10–13 Years

Associations between change in physical activity and seven biological and demographic variables were examined in 20 studies.^18,22–25,27,32,33,35,42,47–52,54,57,58,63^ Previous physical activity^22,25,43^ and self-efficacy^25,37,43^ were consistently positively associated with change in physical activity. There was consistent evidence of no association with change in physical activity for value of health, appearance and achievement,^48,52^ anthropometry,^22,24,26,42,43,46^ parental marital status,^32,39^ parental support,^24,32,39^ smoking,^48,52,59^ barriers to physical activity,^33,42^ parental physical activity attitudes,^26,48^ parental role modeling,^25,37,42,43^ and parental physical activity.^42,48^

### Adolescents Aged 14 Years and Older

Eight studies investigated biological and demographic variables such as gender, ethnicity, and anthropometry.^28,34,38,40,45,53,55,60^ Fourteen psychological factors were examined by nine studies,^30,34,36,38,39,41,53,55,64^ whereas only four behavioral factors and two environmental factors were investigated in four^28,34,36,55^ and two^36,38^ studies, respectively. Higher scores on perceived behavioral control,^36,38,41,53^ social support,^30,39,55^ and self-efficacy^30,38,39,41,55^ measures were consistently associated with smaller declines in physical activity. Change in physical activity was consistently not associated with ethnicity^38,53^ and physical activity attitude.^36,38^

### Study Quality

Seven^6,23,32,33,35,60,62^ of the high-quality studies employed objective measures of physical activity, and high-quality studies were found in all age groups. The association between perceived behavioral control and change in physical activity was supported by a high-quality study^36^ in adolescents. For self-efficacy, high-quality studies^38,43^ seemed to show an association between change in self-efficacy and change in physical activity rather than an association between baseline self-efficacy and change in physical activity.

## Discussion

This review presents a summary of 46 studies assessing the determinants of change in child and adolescent physical activity. Research into determinants of change in physical activity has increased in recent years, as highlighted by the large proportion of studies reported since 2006. In general, a greater number of studies stem from North America and have relied on self-report measures of physical activity. Latterly, an increasing number of studies employed objectively measured physical activity and originate outside North America, for example Australia. A wide range of potential determinants were investigated across age groups, yet individual factors were not consistently investigated across studies, which limited the scope of analyses in this review.

The results from this review confirmed some, but not all, of the previously established correlates of physical activity within the three age groups. Among the younger children, girls consistently reported larger declines in physical activity than boys, which certainly mirrors previously established evidence on correlates of physical activity,^10,11^ and parental marital status was consistently shown not to be associated with change in activity. In children aged 10–13 years, no consistent results were evident for the association between physical activity and gender, whereas results consistently showed no association between gender and change in physical activity in adolescents. It may be possible that the gender differences in children decline with increasing age or maturity.

Self-efficacy was associated with change in physical activity in older children and adolescents; both the strength and the direction of these associations are comparable to results reported in cross-sectional studies.^10,11^ Higher levels of self-efficacy were associated with smaller declines compared to lower levels of self-efficacy. More specifically, a one-unit increase in self-efficacy was positively associated with changes ranging from 0.06 of a MET in a study by Dzewaltowski et al.,^43^ more than 0.15 METs in a study by Dishman et al.^38^ to a change in 1 MET in analyses conducted by Dowda et al.^41^ In these analyses, self-efficacy was constructed from eight items scored on a 5-point scale by Dishman et al.^38^ and Dowda et al.,^41^ whereas Dzewaltowski et al.^43^ measured self-efficacy with multiple items scored on a 6-point scale. Different coding approaches and the use of differing types of self-efficacy constructs may have influenced this range of values.

Not all established correlates could be confirmed longitudinally. A hypothesized inverse association between developmental stage and change in physical activity in children aged 10–13 years, based on evidence from previous studies,^65–67^ could not be confirmed. This finding may be due to differences in the maturity measurement protocols and the imprecision of these measures.

Despite the unclear picture of its association with change in physical activity, maturation may be an important factor to consider when investigating age-related declines in physical activity.^68^ As there is inter-individual variation in the timing and tempo of physical maturation, maturity may directly or indirectly explain inter-individual differences in physical activity decline.^67^ Other factors exhibiting similar associations to previously established cross-sectional results were perceived behavioral control, parental role modeling, parental activity, and barriers for physical activity.^11^

Despite the recent shift in interest toward environmental correlates of physical activity,^9,20^ very few studies investigated environmental factors and change in physical activity in children and adolescents. Only six studies^31,33,36–38,61^ investigated environmental determinants across all age groups, although a wide range of factors was considered. Associations between potential determinants of change in physical activity appeared to be age group–specific or differed by gender. Despite being unable to draw conclusions, there appeared to be a tendency toward an association between smaller declines in physical activity and road length and traffic-regulating features of the environment such as traffic lights or number of speed bumps. Attention should however be given to the statistical model components when comparing results of analyses on environmental determinants of change in physical activity across studies. Based on the ecologic model, direct associations between the more distal environmental factors and change in physical activity may be moderated by more proximal social or psychological factors. The interactions among factors in the different domains of the ecologic model should be given more attention.^69^

Correlates of physical activity have been shown to differ between children and adolescents, yet age group–specific differences in determinants of change in physical activity could not be ascertained because of the low number of studies per age group. However, age group–specific tendencies in research focus and preference could be observed. For instance, only eight studies^6,21,24,29,31,44,46,62^ investigated determinants of change in children aged <10 years. Further, the investigated determinants appeared to focus on biological and demographic factors, with relatively little research on psychological, behavioral, environmental factors and parent–child interaction. This lack of research, both in the investigated domains of potential determinants and the low number of studies available, may be the result of limited availability of appropriate and nonvalidated research instruments to measure physical activity and its determinants.^70,71^ In comparison, research into adolescent determinants of change in physical activity appear to be focused on a wide range of psychological constructs. These age-specific tendencies may reflect immediate research interests and priorities in the respective age groups and highlights areas of interest for future research.

Two types of longitudinal associations were included: predictors of change and correlated changes between the exposure and outcome. Combining both types of association for the purpose of this review did not influence the results. However, some studies investigating self-efficacy, peer support, and perceived peer support reported differences in associations with change in physical activity based on whether the aforementioned factors were modeled as baseline determinants or change variables. In some studies, when modeled as a predictor at baseline these factors showed no association with change in physical activity, yet a positive association was reported for correlated changes between the investigated factor and physical activity. This might hint at a reverse causality between the postulated determinants and change in physical activity. Indeed, this may be an indication of the social and psychological benefits of physical activity, which have previously been reported.^2^ Despite there being similarities between results from both types of analysis and change in physical activity in youth, future research may benefit from exploring both types of association, predictors and correlated changes, separately. This may help ascertain causal pathways and disentangle issues of reverse causality between psychological factors and physical activity.^12^

The included studies were heterogeneous in study design, analysis method, outcome measures, and investigated exposures. For instance, physical activity measurement encompassed both objective and self-report measures of physical activity, but rarely in the same study. In addition, measures of self-reported physical activity ranged from the validated Physical Activity Questionnaire for Adolescents to nonvalidated single-item physical activity instruments. A multitude of physical activity subcomponents were investigated, such as change in sports participation, change in physical activity–related energy expenditure, or change in moderate and vigorous activity. The resulting variation in outcome measures may have contributed to the low number of consistent associations found in this review, as correlates and determinants may differ for various subcomponents of physical activity.

These results emphasize a recent call toward the analysis of specific subcomponents of physical activity in relation to potential correlates and determinants, both in single analyses and systematic reviews.^72^ Further, inconsistent terminology was used across studies and a number of studies relied on nonvalidated constructs. The resulting variation in both measurement and reporting of measured constructs restricts accurate cross-study comparisons, which has previously been highlighted.^69^ Despite this heterogeneity, mean study quality was reasonably high, particularly among more recent studies. The index used in this analysis reflects rigorous study design and analysis methods, as well as high-quality reporting of the study results.

### Implications for Future Research

This review has highlighted two modifiable factors from a single domain that appear to be consistently associated with change in physical activity: self-efficacy and perceived behavioral control. These factors may be a focus when developing future physical activity interventions. However, in light of recent interest in multi-component interventions, more evidence is needed encompassing all domains of the ecologic model—especially regarding the potential interaction among its differing domains.^69^ Research should aim to incorporate high-quality and validated measures for both exposure variables and outcome measures. This should include objective measures of physical activity where possible and use previously validated questionnaires to assess the investigated determinants. More research is needed in all age groups—especially in younger children and adolescents, as the majority of studies have been conducted in those aged 10–13 years.

Future research should be directed toward comprehensive assessments of determinants and mediators of change in physical activity within each category, aiming to build the evidence base relating to environmental determinants of change in physical activity. Research should include both subjective perceptions as well as objective measurements of the environment. It should also address the context of physical activity,^72^ such as leisure-time, school-based, or weekend physical activity, and potential differences in the association between objective and subjectively measured variables with change in physical activity. In addition, investigation into determinants of change should take specific physical intensities such as minutes spent in moderate or vigorous physical activity into account.

### Strengths and Limitations of the Systematic Review

This review identified a large number of potential studies obtained from literature searches in a wide range of databases. The broad definition of search terms and systematic search strategy should have enabled this review to detect as many potential studies as possible. However, some studies may have been overlooked because of misleading key words, titles, or abstracts. As this review was restricted to published studies only, publication bias may be present.

The heterogeneity in study samples, exposure and outcome measures included in this review limited interpretation and meant that it was not possible to meta-analyze the results. The semi-quantitative reporting in this review provides only a somewhat arbitrary classification of the associations with focus on the direction rather than on the strength of the association.^10^ The observed heterogeneity led to a rudimentary grouping of investigated exposures into their overarching constructs and the investigation of overall change physical activity as the outcome of interest. A number of analyses have drawn data from the same cohort studies, for example the Children Living in Active Neighborhoods^24,31,33^ or Lifestyle Education for Activity Program^38,39,41^ studies, which may introduce bias into the analysis sample.

As the scope of this review primarily was to collate the existing evidence base, a potential weighting of scores was not considered. In two instances, a potential determinant was investigated as a predictor of change in physical activity by two analyses^38,39^ drawing on one data set. Results were unaffected by the exclusion of the overlapping results. In two cases, the direction of the association was inferred according to the most likely outcome; this did not have an impact on the conclusions drawn from this review.

## Conclusion

This review presents a synthesis of the literature assessing the determinants of change in physical activity among children and adolescents. Consistent associations for self-efficacy in children aged 10–13 years and self-efficacy and perceived behavioral control in adolescents complemented previously established correlates of physical activity in direction and strength. However, other hypothesized determinants such as developmental stage could not be fully confirmed. Further, inconclusive associations were reported for a large proportion of the potential determinants investigated. Age group–specific trends highlighted areas of interest and outlined future research needs. The heterogeneity in study samples, exposure, and outcome measures limit our ability to draw conclusions in this review and highlights the need for further research. Future research should aim to comprehensively assess potential determinants of physical activity in youth across all domains of the ecologic model, utilizing validated constructs and objectively measured physical activity—especially in younger children.

## Figures and Tables

**Table 1:** Child and adolescent studies categorized by the baseline age of the included sample, publication year of the study, and analysis method employed

	References
**Sample baseline age (years)**	
≤9	6, 21, 24, 29, 31, 44, 46, 62
10–13	18, 22, 23, 24, 25, 26, 27, 31, 32, 33, 35, 42, 43, 47, 48, 49, 50, 51, 52, 54, 56, 57, 58, 59
≥14	28, 30, 34, 36, 38, 39, 40, 41, 45, 53, 55, 60
**Publication year**	
<2000	18, 22, 39, 40, 45, 46, 58
2000–2005	23, 25, 29, 34, 52, 53, 55, 63, 64
2006–2008	6, 26, 27, 35, 36, 38, 41, 42, 43, 47, 48, 49, 50, 51, 54, 56, 57, 59, 60
>2009	21, 24, 28, 30, 31, 32, 33, 39, 44, 62
**Country of study origin**	
U.S.	6, 18, 21, 22, 26, 29, 35, 38, 39, 41, 42, 43, 46, 47, 48, 50, 51, 52, 53, 54, 55, 59, 63
Canada	25, 28, 34, 44, 58
Europe	23, 27, 32, 36, 40, 45, 49, 53, 56, 57, 60, 62
Australia	24, 31, 33
Hong Kong	30
**Physical activity measurement**	
Self-report	18, 21, 22, 25, 26, 27, 29, 30, 34, 36, 37, 38, 39, 40, 41, 43, 44, 45, 47, 48, 49, 50, 52, 53, 54, 55, 56, 57, 58, 59, 64
Objective measures	6, 23, 32, 46, 51, 61
Both self-report and objective	24, 28, 31, 33, 35, 42, 60, 62, 63
**Methodologic quality of the included studies (Quality Score 0–5)**	
0–1	22, 26, 27, 38, 42
2–3	21, 24, 25, 29, 30, 31, 34, 39, 41, 43, 44, 45, 47, 48, 50, 51, 52, 53, 56, 57, 61
4–5	6, 18, 23, 32, 33, 35, 36, 40, 49, 54, 55, 58, 59, 60, 62
**Follow-up time (years)**	
≤1	18, 28, 29, 32, 34, 36, 49, 53, 55, 58, 59, 62, 64
2–3	23, 24, 25, 31, 33, 35, 37, 38, 39, 40, 41, 42, 43, 45, 48, 51, 52, 54, 56, 57, 60, 61
≥4	6, 21, 22, 26, 30, 44, 46, 47, 50, 63

**Table 2:** Determinants of change in physical activity in children and adolescents

Factors	Children aged ≤9 years			Children aged 10–13 years			Children aged ≥14 years		
	References	Associations	Summary	References	Associations	Summary	References	Associations	Summary
**Biological and demographic**									
Gender	6, 21, 24, 61	—	—	18, 22, 23, 24, 27, 32	—	??	28, 40(f), 45	0	*??*
				48, 50, 51, 52	0				
Age (years)/grade				23, 48, 54	—	??	28	—	*??*
				25(m), 25(f), 47, 51	0		55(f)	0	
Developmental stage				23, 35, 42(m), 63(f)	—	??			
				42(f), 49(f), 48, 50	0				
Anthropometry	6, 61(f)	—	??	32, 57, 63(f)	—	00	34(f), 55(f)	0	*??*
	61(m)	0		50	?				
				23, 25(m), 25(f), 27(m), 27(f), 47, 48, 51	0				
SES	6, 44(m)	++	??	25(f), 27(f), 50	++	??	60	++	*??*
	44(f)	—		32, 33(m)	—		24, 53(f)	0	
	21, 24,61(m), 61(f)	0		24, 25(m), 27(m), 33(f), 42(m), 42(f)	0		45	?	
Ethnicity	61(m), 61(f)	0	??	25(m), 27, 58	—	??	38(f), 53(f)	0	00
				18, 25(f), 42(m), 42(f), 50	0		60	?	
Region	6, 28(m), 28(f)	0	??	25(m), 50	?	??			
				25(f)	0				
Urban/rural	44(m), 44(f)	0	??						
Area deprivation	32	0	??						
Weekend days	62	—	??						
**Psychological**									
Preference for physical activity	61(f)	++	??						
	61(m)	?							
Perceived competence	29(f), 61(m)	++	??	35, 51	—	??	64(m)	++	*00*
	61(f)	0		18	?		55(f), 64(f)	0	
				31	0				
Physical perception	61(m), 61(f)	0	??	18, 35, 49(f), 51	?	??	34(f)	?	*??*
							55(f)	0	
Self-worth	61(m), 61(f)	0	??	18	?	??	34(f)	++	*??*
							55(f)	+	
Self-acceptance	61(m), 61(f)	0	??				55(f)	0	*??*
Satisfaction				22	0	??	34(f), 55(f)	0	*??*
Reward				22	0	??			
Self-esteem				48	0	??	34(f)	0	*??*
Self-efficacy				25(m), 25(f), 37(m), 37(f), 43	++	*++*	30, 38(f), 39(f), 41(f), 55(f)	++	*++*
				42(m), 42(f)	?		53(f)	0	
Goal-setting							41(f)	++	*??*
Depressive symptoms				52	—	??	55(f)	0	*??*
				35	?				
Physical activity attitude	61(m), 61(f)	0	??	18	0	??	41(f)	++	00
							36, 38(f)	0	
Physical education attitude	61(m)	+	??						
	61(f)	0							
Perceived behavioral control				58	++	??	36, 38(f), 41(f), 53(f)	++	*++*
Intention	61(m), 61(f)	0	??	58	++	??	38(f)	++	*??*
							36	0	
Enjoyment of physical activity				37(m), 37(f)	0	??	64(m)	++	*??*
							55(f), 64(f)	0	
Benefits of physical activity							55(f)	0	*??*
Value of health, appearance, achievements				54	++	00			
				48, 52	0				
Maturity fears				35	?	??			
Exercise knowledge				37(m), 37(f)	++	??			
Interest in sports media				37(m), 37(f)	0	??			
**Behavioral**									
Vigorous physical activity							28	–	*??*
Previous physical activity				22, 25(f), 43	++	++	36	++	*??*
				25(m)	0				
Alcohol consumption				52	0	??			
Smoking status				54	—	00			
				25(m), 25(f), 52, 59(f)	0				
Sedentary behavior	61(m), 61(f)	0	??	37(m), 37(f), 54, 56	—	??	55(f)	0	*??*
				25(m), 25(f), 59(f)	0				
Dietary habits							34(f)	0	*??*
Participation In sports teams outside school				25(m), 25(f)	0	??			
**Sociocultural**									
Support for physical activity							30, 39(f), 55(f)	++	*++*
Parental attitudes toward physical activity				26(m), 26(f), 48	0	??			
Parental/family support	29(f)	++	??	26(m), 26(f)	?	00	41(f)	++	*??*
	61(m), 61(f)	0		25(m), 25(f), 33(m), 33(f), 37(m), 37(f), 42(m), 42(f)	0				
Parental role modeling				25(m), 25(f), 37(m), 37(f), 42(m), 42(f), 43	0	00			
				33(m), 33(f)	?				
Parental co-participation in physical activity				33(f)	++	??			
				33(m)					
Parental self-efficacy				37(f)	++	??			
				37(m)	?				
Parental physical activity	61(m)	++	??	42(m), 42(f), 48	0	00	64(f)	++	*??*
	61(f)	0		37(m), 37(f)	?		64(m)	0	
Parental weight status				63(f)	0	??			
Sibling physical activity				33(m)	++	??			
				33(f)	0				
Number of siblings				33(f)	++	??			
				33(m)	0				
Peer attitudes				48	0	??	64(m), 64(f)	++	*??*
Peer social support				57	++	??			
				37(f)	?				
				37(m), 42(m), 42(f)	0				
Peer physical activity				42(m)	++	??			
				42(f)	?				
Parental marital status	44(m), 44(f), 61(m), 61(f)	0	00	33(m), 33(f), 42(m), 42(f)	0	00			
Barriers				37(f)	?	00	64(f)	—	*??*
							64(m)	0	
				37(m), 48	0		55(f)	?	
Social group subjective norm							36, 38(f)	0	*??*
Rules for physical activity and sedentary activities				33(f)	++	??			
				33(m)	0				
**Physical environmental**									
Availability of physical activity infrastructure/equipment	31(m)	?	??	33(m), 33(f), 37(m), 37(f)	0	00	38(f)	++	*??*
				36	?				
Neighborhood safety	61(m), 61(f)	0	??						
Aesthetics in the environment							36	0	*??*
Distance to school	31(m)	++	??	33(m), 33(f)	0	??			
	31(f)	—							
Road length	31(m)	++	??	31(f)	+	??			
	31(f)	0	??	31(m), 33(m), 33(f)	0				
Road traffic	31(m)	—	??	33(m)	—	??			
	31(f)	?		31(m), 31(f), 33(f)	0				
Road characteristics	31(m), 31(f)	?	??	33(m), 33(f)	0	??			

*Note:* Associations: ++, positive (*p*≤0.05); +, positive (*p*<0.1); −−, negative (*p*≤0.05); –, negative (*p*<0.1); ?, indeterminate; 0, no association. Summary Associations: ++, positive; – –, negative; 00, no association; ??, indeterminate. Studies reporting separate findings for boys and girls were indicated with m (boys) and f (girls).
