# Supplementary material for: Determinants of Change in Physical Activity in Children and Adolescents: A Systematic Review
Source: Am J Prev Med. 2011 Jun;40(6):645–58. doi: 10.1016/j.amepre.2011.02.025 (PMC3100507; doi:10.1016/j.amepre.2011.02.025)
Supplement: Appendix A and B [file mmc1.pdf]

Determinants of Change in Physical Activity in Children and Adolescents
A Systematic Review

Christopher Craggs, Msc, Kirsten Corder, PhD, Esther M.F. van Sluijs, PhD, Simon J. Griffin, DM

Appendix A

Criteria used to assess study quality of all studies included in the review.

A	Adequate description of study population<br>Adequate reporting of baseline and follow-up participant characteristics, and physical activity at baseline and follow-up<br><br>(Positive if information on baseline characteristics—level and/or distribution—of the exposure variables included in the analyses were provided, if the study setting was reported, and if levels of physical activity were reported for all strata at baseline and follow-up)
B	Adequate follow-up and information on representativeness of the study sample at baseline and follow-up, including information on potential systematic dropout<br><br>(Positive if follow-up rates were documented and if information on characteristics of responders and nonresponders at baseline at follow-up were presented and potential differences statistically tested)
C	Validated measure of physical activity and adequate physical activity measurement<br><br>(Positive if the same measure of physical activity was employed at both baseline and follow-up and if this method of measuring physical activity was validated and reported/referred to in the relevant age groups at both baseline age and follow-up age)
D	Validated measurement of exposure variables<br><br>(Positive if standardized questionnaires and/or validated measures were used to assess exposure variables and were reported/referred to)
E	Potential confounders and sources of bias considered in analyses<br><br>(Positive if statistical analyses have been adjusted for known or potential confounders; for instance, if multilevel modeling was employed to account for potential clustering at group level)

Scoring:
Positive
Negative
Not described or insufficient information provided

Appendix B

Summary characteristics of the studies included in the systematic review

Study	Country	Study characteristics: age (years); *n* (% girls); setting	Period of change (years); (# of measure-ments)	Physical activity measurement	Modeling of change	Change measured	Investigated exposures
Alderman (2010)[1]	U.S.	5.3 (SD 0.2); 96; university-based preschool movement program	1–9	Self-report	Difference testing	Change in physical activity	Gender
Anderson (1986)[2]	U.S.	10–12; 238; School district in Iowa	5 (2)	Self-report (1-year recall)	Regression model	Change in sports participation	Gender, previous physical activity, satisfaction, reward
Armstrong (2000)[3]	UK	11.1 (0.4); 202 (48); 15 state schools in Exeter, UK	2 (3)	Heart rate monitoring	Multilevel regression model	Change in MVPA	Gender, BMI/anthropometry, developmental stage, age
Ball (2009)[4]	Australia	5–6 and 10–12; 1215 (53); 19 public elementary schools	3 (2)	Accelerometry	Linear regression model	Change in physical activity, MPA, VPA	Gender, SES
Barnett (2002)[5]	Canada	9–11; 2318; 16 schools in Montreal, Canada	2 (3)	Self-report (7-day recall)	Multilevel regression model	Decline to less than one activity per day	Age, grade, BMI, ethnicity, smoking status, sedentary behavior, previous physical activity, region, SES, self-efficacy, parental/family support, parental role modeling
Bauer (2008)[6]	U.S.	12.8 (SD 0.8); 5287; 31 middle and high schools in Minnesota	5 (2)	Self-report (7 day recall).	Regression model	Change in MVPA	SES, ethnicity, parental/family support, parental attitudes toward physical activity
Brodersen (2007)[7]	UK	11–12; 474; 36 randomly selected schools in London, UK	4 (5)	Self-report (7-day recall)	Multilevel regression model	Change in average days spent in VPA	Gender, ethnicity, SES
Bruner (2009)[8]	Canada	15.3 (SD 1.3); 40; mid-size public school	8 months (5)	Accelerometry	Generalized estimating equation	Change in physical activity	Gender, grade, VPA
Butcher (2001)[9]	U.S.	9.1; 705 (100); seven elementary schools	1 (2)	Self-report	Regression model	Change in sports participation	Perceived competence, parental/family support
Callaghan (2010)[10]	Hong Kong	16; 1055; public secondary schools	5 (20)	Self-report	Multivariate ANOVA	Change in exercise behavior	Self-efficacy, social liberation

Study	Country	Study characteristics: age (years); *n* (% girls); setting	Period of change (years); (# of measure-ments)	Physical activity measurement	Modeling of change	Change measured	Investigated exposures
Carver (2009)[11]	Australia	5–6 and10–12; 1215 (53); 19 public elementary schools	3 (2)	Accelerometry	Linear regression model	Change in MVPA	Age, gender, road traffic, physical activity infrastructure, road length, intersection density, number of speed bumps, number of traffic lights, local road index
Corder (2010)[12]	UK	10.2 (SD 0.3); 884 (58); 92 primary schools in Norfolk, UK	1 (2)	Accelerometry	Multilevel linear regression models	Change in total physical activity, MVPA	Baseline physical activity, gender, SES, body fat %, Index of Multiple Deprivation
Crawford (2010)[13]	Australia	5–6 and10–12; 1215 (53); 19 public elementary schools	3 (2)	Accelerometry	Linear regression model	Change in MVPA	BMI, SES, road traffic, number of siblings, sibling physical activity, parental role modeling, number of rules for physical activity and sedentary behavior, parental marital status, parental support, parental physical activity participation, physical activity infrastructure, distance to school, road length
Crocker (2003)[14]	Canada	15–16; 178 (100); city and town (32%) high schools in Saskatoon, Canada	1 (2)	PAQ-A (7-day recall)	Regression model	Change in physical activity	BMI, physical perception, self-worth, satisfaction, self-esteem, dietary habits
Davison (2007)[15]	U.S.	11.3 (SD 0.3); 221 (60); 10-year longitudinal study in five- county radius in Pennsylvania	2 (2)	Self-report (Children's Physical Activity Scale) and accelerometry	Structural equation model	Change in MVPA	Developmental stage, perceived competence, physical perception, depressive symptoms, maturity fears

Study	Country	Study characteristics: age (years); *n* (% girls); setting	Period of change (years); (# of measure-ments)	Physical activity measurement	Modeling of change	Change measured	Investigated exposures
de Bruijn (2006)[16]	Nether-lands	15.1 (SD 1.9); 221 (60); 23 general practitioners from nine family practice centers in Eindhoven, Netherlands	4 months (2) [May–November]	Self-report	Structural equation model	Change in physical activity	Previous physical activity, physical activity attitude, perceived behavioral control, intention, social subjective norm, availability of physical activity infrastructure, neighborhood safety, aesthetics in the environment
DiLorenzo (1998)[17]	U.S.	11.2 (SD 0.7); 242 (50); two randomly selected community schools	3 (2)	Self-report (physical activity)	Regression model	Change in daily MET tasks	Self-efficacy, enjoyment of physical activity, exercise knowledge, interest in sports media, sedentary behavior, parental/family support, parental role modeling, parental self-efficacy, peer social support, parental physical activity, barriers, availability of physical activity equipment
Dishman (2006)[18]	U.S.	14.1 (SD 0.6); 1037 (100); 22 public high schools in South Carolina	3 (2)	3DPAR (3-day recall)	Structural equation model	Change in 3-day MET tasks	Ethnicity, physical activity attitude, satisfaction, social subjective norm, perceived behavioral control, self-efficacy, goal setting
Dishman (2009)[19]	U.S.	14.1 (SD 0.6); 1037 (100); 22 public high schools in South Carolina	3 (2)	3DPAR (3-day recall)	Structural equation model	Change in physical activity	Self-efficacy, social support
Dovey (1998)[20]	New Zealand	15; 421 (100); cohort of live births at a Dunedin hospital, New Zealand	3 (2)	Self-report (1 year recall, by month)	Repeated measures ANOVA	Decline in time spent in physical activity	Gender
Dowda (2007)[21]	U.S.	13.6 (SD 0.6); 421 (100); 24 high schools in South Carolina	5 (3)	3DPAR	Growth curve model	Change in MET tasks	Ethnicity, SES, physical activity attitude, parental/family support, self-efficacy, availability of sports equipment

Study	Country	Study characteristics: age (years); *n* (% girls); setting	Period of change (years); (# of measure-ments)	Physical activity measurement	Modeling of change	Change measured	Investigated exposures
Duncan (2007)[22]	U.S.	12.1 (SD 1.63); 371 (50.1); 58 neighborhoods in the Pacific North West	3 (4)	Three self-report items (two taken from Youth Risk Behavior Survey - YRBS) and pedometer	Latent growth curve model	Change in physical activity	BMI, developmental stage, SES, ethnicity, self-efficacy, peer social support, peer physical activity
Dzewaltowski (2007)[23]	U.S.	12.4; 629 (53); 16 middle schools in Kansas	2 (3)	3DPAR	Latent growth curve model	Change in MVPA	Self-efficacy
Findlay (2009)[24]	Canada	4–11; 8817 (51); national longitudinal survey	7 (7)	Self-report	Group based trajectory modeling	Change in physical activity	SES, parental marital status, urban–rural status
Fuchs (1988)[25]	West Germany	13.3 (SD 0.9); 932 (46); 22 schools in Berlin and Bremen, Germany	2 (3)	1-year physical activity recall	Wilcoxon test for trend	Change in total physical activity, MVPA, and VPA	Gender, SES
Garcia (1998)[26]	U.S.	10–12; 132 (58); junior high schools	1 (2)	7-day recall (CAAL-Child/Adolescent Activity Log)	Structural equation model	Change in physical activity	Gender, ethnicity, physical activity attitude
Goran (1998)[27]	U.S.	5.5 (girls, SD 0.9); 5.3 (boys, SD 0.9); 22 (50); cohort in Burlington, VT	5 (3)	Doubly labeled water over 14 days	ANOVA	Change in total energy expenditure, AEE, activity (hours/day)	Gender
Hampson (2007)[28]	U.S.	9.5; 846 (50); 15 elementary schools in one school district in Oregon	7 (5)	Self-report	Latent growth curve model	Change in athleticism	Age, gender, BMI, physical activity attitude
Kahn (2008)[29]	U.S.	10–16; 12,812 (57); children from 50 states in the U.S., offspring from the Nurses Health Study	2 (3)	1-year physical recall	Mixed regression model	Change in MVPA	Age, gender, BMI, self-esteem, value of health and appearance, peer attitudes, parental attitudes to physical activity, parental physical activity, social group subjective norm
Knowles 2009)[30]	UK	11.8 (0.4); 204; 17 primary schools and six secondary schools in Edinburgh, UK	1 (2)	PAQ-C (7-day recall)	Linear regression	Change in physical activity	Developmental stage, physical self-perceptions
McMurray (2008)[31]	U.S.	10; 765 (49); 21 elementary schools and 16 high schools in North Carolina	5 (2)	Self-report (7-day recall)	ANOVA	Decline in total physical activity, MPA and VPA	Gender, BMI, ethnicity, region, developmental stage, SES

Study	Country	Study characteristics: age (years); *n* (% girls); setting	Period of change (years); (# of measure-ments)	Physical activity measurement	Modeling of change	Change measured	Investigated exposures
Morgan (2008)[32]	U.S.	6–12; 257; two public elementary schools, U.S.	27 months (2)	Pedometers (4 days—elementary students; 8 days—junior high)	Regression model	Change in steps taken	Age, gender, BMI, perceived competence, physical self-perception
Motl (2004)[33]	U.S.	12.7 (SD 0.4); 3878 (49); 16 schools in Minneapolis, MN	2 (3)	Self-report	Latent growth curve model	Change in physical activity	Gender, depressive symptoms, alcohol consumption, smoking status
Motl (2005)[34]	U.S.	13.6 (SD 0.6); 856 (100); 24 high schools in South Carolina	1 (2)	3DPAR	Structural equation model	Change in physical activity, MPA and VPA	Ethnicity, self-efficacy, perceived behavioral control
Motl (2006)[35]	U.S.	12.7 (SD 0.4); 3878 (49); 16 schools in Minneapolis, MN	2 (3)	Self-report	Latent growth curve model	Change in physical activity	Gender, grade, value of health and appearance, sedentary behavior, smoking status
Nader (2008)[36]	U.S.	9.0 (SD 0.03); 771 (51); recruitment at community hospitals at 10 university data collection sites, U.S.	6 (4)	Accelerometry	Growth curve model	Change in weekday physical activity	Gender, BMI, SES, ethnicity, region
Neumark-Sztainer (2003)[37]	U.S.	15.4 (SD 1.1); 201 (100); six high schools (three control schools) in the Minneapolis/St. Paul, MN area	1 (3)	Self-report (7-day recall)	Regression model	Change in MVPA, MPA, and VPA	Self-acceptance, satisfaction, barriers, sedentary behavior, physical perception, depressive symptoms, perceived competence, self-efficacy, enjoyment of physical activity, benefits of physical activity, parental attitudes to physical activity, parental marital status
Raudsepp (2008)[38]	Estonia	12.6 (SD 0.5); 193 (100); four high schools in Tartu, Estonia	1.75 (4)	3DPAR	Latent growth curve model	Combined change in MET tasks, MVPA, and VPA	BMI, peer social support
Raudsepp (2008)[39]	Estonia	12.3 (SD 0.4); 345 (50); four high schools in Tartu, Estonia	1.75 (4)	3DPAR	Latent growth curve model	Change in MET tasks, MVPA, and VPA	Sedentary behavior
Rhodes (2006)[40]	Canada	11.2 (SD 0.6); 364 (51); 10 schools in British Columbia, Canada	1 (2)	Self-report	Structural equation model	Change in physical activity	Intention, perceived behavioral control

Study	Country	Study characteristics: age (years); *n* (% girls); setting	Period of change (years); (# of measure-ments)	Physical activity measurement	Modeling of change	Change measured	Investigated exposures
Robinson (1993)[41]	U.S.	12.4 (SD 0.7); 674 (100); four middle schools in northern California	1 (3)	Self-report	Regression model	Change in physical activity	Sedentary behavior
Sagatun (2008)[42]	Norway	15–16; 2489 (55%); 32 secondary schools in Oslo	3 (2)	Accelerometry and self-report	ANOVA and linear regression model	Change in leisure time activity	Ethnicity, SES
Sallis (1999)[43]	U.S.	9.5 (SD 0.6); 732 (51); seven suburban public schools in San Diego	20 months (2)	Accelerometer measurement, child self-report, and parent report	Regression model	Change in physical activity index	BMI, anthropometry, SES, ethnicity, preference for physical activity, perceived competence, physical perception, self-worth, self-acceptance, physical activity attitude, PE attitude, Intention, sedentary behavior, parental/family support, parental physical activity, parental marital status, neighborhood safety
Sigmund (2009)[44]	Czech Republic	5–7; 208; 11 kindergarten schools in Marovia	1 (2)	Accelerometry and self-report	ANOVA	Change in AEE	Days of the week
Spadano (2005)[45]	U.S.	9.9 (SD 0.4); 28 (100); recruitment in Cambridge, MA and Somerville, MA public schools and Massachusetts Institute of Technology summer camp	5 (3)	Doubly labeled water and indirect calorimetry	Mixed-model repeated measure analyses	Change in AEE	BMI/anthropometry, ethnicity, developmental stage, parental weight status
Viira (2003)[46]	Estonia	13.1 (boys, SD 0.2); 13.0 (girls, SD 0.4); 197 (53); middle and high schools in Estonia	1 (2)	Self-report (7-day recall)	Regression model	Change in MVPA	Peer attitudes, enjoyment of physical activity, physical perception, barriers, parental physical activity

If the mean age of the sample was not reported, the reported age group or age range was included.

AEE, activity energy expenditure; MPA, moderate physical activity; MVPA, moderate-to-vigorous physical activity; N/A, data were not reported; PAQ, physical activity questionnaire; PE, physical education; VPA, vigorous physical activity; 3DPAR, 3-day physical activity recall

References for Appendix B

1. Alderman BL, Benham-Deal TB, Jenkins JM. Change in parental influence on children's physical activity over time. J Phys Act Health 2010;7(1):60–7.
2. Anderson DF, Lorenz FO, Pease DG. Prediction of present participation from children's gender, past participation, and attitudes: a longitudinal analysis. Sociol Sport J 1986;3(2):101–11.
3. Armstrong N, Welsman JR, Kirby BJ. Longitudinal changes in 11–13-year-olds' physical activity. Acta Paediatr 2000;89(7):775–80.
4. Ball K, Cleland VJ, Timperio AF, Salmon J, Crawford DA. Socioeconomic position and children's physical activity and sedentary behaviors: longitudinal findings from the CLAN study. J Phys Act Health 2009;6(3):289–98.
5. Barnett TA, O'Loughlin J, Paradis G. One- and two-year predictors of decline in physical activity among inner-city schoolchildren. Am J Prev Med 2002;23(2):121–8.
6. Bauer KW, Nelson MC, Boutelle KN, Neumark-Sztainer D. Parental influences on adolescents' physical activity and sedentary behavior: longitudinal findings from Project EAT-II. Int J Behav Nutr Phys Act 2008;5.
7. Brodersen NH, Steptoe A, Boniface DR, Wardle J. Trends in physical activity and sedentary behaviour in adolescence: ethnic and socioeconomic differences. Br J Sports Med 2007;41(3):140–4.
8. Bruner MW, Chad KE, Beattie-Flath JA, et al. Examination of physical activity in adolescents over the school year. Pediatr Exerc Sci 2009;21(4):421–35.
9. Butcher J, Sallis JF, McKenzie TL, Alcaraz JE. A longitudinal study of children's participation in organized sport and instructional programs. AVANTE 2001;7(1):86–96.
10. Callaghan P, Khalil E, Morres I. A prospective evaluation of the Transtheoretical Model of Change applied to exercise in young people. Int J Nurs Stud 2010;47(1):3–12.
11. Carver A, Timperio A, Hesketh K, Crawford D. Are safety-related features of the road environment associated with smaller declines in physical activity among youth? J Urban Health 2009;87(1):29–43.
12. Corder K, van Sluijs EM, Ekelund U, Jones AP, Griffin SJ. Changes in children's physical activity over 12 months: longitudinal results from the SPEEDY study. Pediatrics 2010;126(4):e926–35.
13. Crawford D, Cleland V, Timperio A, et al. The longitudinal influence of home and neighbourhood environments on children's body mass index and physical activity over 5 years: the CLAN study. Int J Obes 2010;34(7):1177–87.
14. Crocker P, Sabiston C, Forrestor S, Kowalski N, Kowalski K, McDonough M. Predicting change in physical activity, dietary restraint, and physique anxiety in adolescent girls: examining covariance in physical self-perceptions. Can J Public Health 2003;94(5):332–7.
15. Davison KK, Werder JL, Trost SG, Baker BL, Birch LL. Why are early maturing girls less active? Links between pubertal development, psychological well-being, and physical activity among girls at ages 11 and 13. Soc Sci Med 2007;64(12):2391–404.
16. de Bruijn GJ, Kremers SPJ, Lensvelt-Mulders G, de Vries H, van Mechelen W, Brug J. Modeling individual and physical environmental factors with adolescent physical activity. Am J Prev Med 2006;30(6):507–12.
17. DiLorenzo TM, Stucky-Ropp RC, Vander Wal JS, Gotham HJ. Determinants of exercise among children. II. A longitudinal analysis. Prev Med 1998;27(3):470–7.

18. Dishman RK, Saunders RP, Felton G, Ward DS, Dowda M, Pate RR. Goals and intentions mediate efficacy beliefs and declining physical activity in high school girls. Am J Prev Med 2006;31(6):475–83.
19. Dishman RK, Saunders RP, Motl RW, Dowda M, Pate RR. Self-efficacy moderates the relation between declines in physical activity and perceived social support in high school girls. J Pediatr Psychol 2009;34(4):441–51.
20. Dovey SM. Continuity and change in sporting and leisure time physical activities during adolescence. Br J Sports Med 1998;32(1):53–57.
21. Dowda M, Dishman RK, Pfeiffer KA, Pate RR. Family support for physical activity in girls from 8th to 12th grade in South Carolina. Prev Med 2007;44(2):153–9.
22. Duncan SC, Duncan TE, Strycker LA, Chaumeton NR. A cohort-sequential latent growth model of physical activity from ages 12 to 17 years. Ann Behav Med 2007;33(1):80–9.
23. Dzewaltowski DA, Karteroliotis K, Welk G, Johnston JA, Nyaronga D, Estabrooks PA. Measurement of self-efficacy and proxy efficacy for middle school youth physical activity. J Sport Exerc Psychol 2007;29(3):310–32.
24. Findlay LC, Garner RE, Kohen DE. Children's organized physical activity patterns from childhood into adolescence. J Phys Act Health 2009;6(6):708–15.
25. Fuchs R, Powell KE, Semmer NK, Dwyer JH, Lippert P, Hoffmeister H. Patterns of physical activity among German adolescents: the Berlin-Bremen Study. Prev Med 1988;17(6):746–63.
26. Garcia AW, Pender NJ, Antonakos CL, Ronis DL. Changes in physical activity beliefs and behaviors of boys and girls across the transition to junior high school. J Adolesc Health 1998;22(5):394–402.
27. Goran MI, Gover BA, Nagy TR, Johnson RK. Developmental changes in energy expenditure and physical activity in children: evidence for a decline in physical activity in girls before puberty. Pediatrics 1998;101(5):887–91.
28. Hampson SE, Andrews JA, Peterson M, Duncan SC. A cognitive-behavioral mechanism leading to adolescent obesity: children's social images and physical activity. Ann Behav Med 2007;34(3):287–94.
29. Kahn JA, Huang B, Gillman MW, et al. Patterns and determinants of physical activity in U.S. adolescents. J Adolesc Health 2008;42(4):369–77.
30. Knowles A-M, Niven AG, Fawkner SG, Henretty JM. A longitudinal examination of the influence of maturation on physical self-perceptions and the relationship with physical activity in early adolescent girls. J Adolesc 2009;32(3):555–66.
31. McMurray RG, Harrell JS, Creighton D, Wang Z, Bangdiwala SI. Influence of physical activity on change in weight status as children become adolescents. Int J Pediatr Obes 2008;3(2):69–77.
32. Morgan CF, Graser SV, Pan Grazi RP. A prospective study of pedometer-determined physical activity and physical self-perceptions in children. Res Q Exerc Sport 2008;79(2):133–40.
33. Motl RW, Birnbaum AS, Kubik MY, Dishman RK. Naturally occurring changes in physical activity are inversely related to depressive symptoms during early adolescence. Psychosom Med 2004;66(3):336–42.
34. Motl RW, Dishman RK, Ward DS, et al. Comparison of barriers to self-efficacy and perceived behavioral control for explaining physical activity across 1 year among adolescent girls. Health Psychol 2005;24(1):106–11.
35. Motl RW, McAuley E, Birnbaum AS, Lytle LA. Naturally occurring changes in time spent watching television are inversely related to frequency of physical activity during early adolescence. J Adolesc 2006;29(1):19–32.

36. Nader PR, Bradley RH, Houts RM, McRitchie SL, O'Brien M. Moderate-to-vigorous physical activity from ages 9 to 15 years. JAMA 2008;300(3):295–305.
37. Neumark-Sztainer D, Story M, Hannan PJ, Tharp T, Rex J. Factors associated with changes in physical activity: a cohort study of inactive adolescent girls. Arch Pediatr Adolesc Med 2003;157(8):803–10.
38. Raudsepp L, Neissaar I, Kull M. Longitudinal stability of sedentary behaviors and physical activity during early adolescence. Pediatr Exerc Sci 2008;20(3):251–62.
39. Raudsepp L, Viira R. Changes in physical activity in adolescent girls: a latent growth modelling approach. Acta Paediatr 2008;97(5):647–52.
40. Rhodes RE, Macdonald HM, McKay HA. Predicting physical activity intention and behavior among children in a longitudinal sample. Soc Sci Med 2006;62(12):3146–56.
41. Robinson TN, Hammer LD, Killen JD, et al. Does television viewing increase obesity and reduce physical activity? Cross-sectional and longitudinal analyses among adolescent girls. Pediatrics 1993;91(2):273–80.
42. Sagatun A, Kolle E, Anderssen SA, Thoresen M, Sogaard AJ. Three-year follow-up of physical activity in Norwegian youth from two ethnic groups: associations with socio-demographic factors. BMC Public Health 2008;8:419.
43. Sallis JF, Alcaraz JE, McKenzie TL, Hovell MF. Predictors of change in children's physical activity over 20 months. Variations by gender and level of adiposity. Am J Prev Med 1999;16(3):222–9.
44. Sigmund E, Sigmundovaj D, El Ansari W. Changes in physical activity in pre-schoolers and first-grade children: longitudinal study in the Czech Republic. Child Care Health Dev 2009;35(3):376–82.
45. Spadano JL, Bandini LG, Must A, Dallal GE, Dietz WH. Longitudinal changes in energy expenditure in girls from late childhood through midadolescence. Am J Clin Nutr 2005;81(5):1102–9.
46. Viira R, Raudsepp L. Psychosocial correlates of physical activity among seventh through eighth grades. J Hum Mov Stud 2003;44(6):501–17
